# Calreticulin promotes EGF-induced EMT in pancreatic cancer cells via Integrin/EGFR-ERK/MAPK signaling pathway

**DOI:** 10.1038/cddis.2017.547

**Published:** 2017-10-26

**Authors:** Weiwei Sheng, Chuanping Chen, Ming Dong, Guosen Wang, Jianping Zhou, He Song, Yang Li, Jian Zhang, Shuangning Ding

**Affiliations:** 1 Department of Gastrointestinal Surgery, the First Hospital, China Medical University, Shenyang 110001, China; 2 Department of Clinical Laboratory, the Sixth Peoples’ hospital of Shenyang, Shenyang 110003, China; 3 Department of Cell Biology, China Medical University, Shenyang 110013, China; 4 Department of Endocrinology and Metabolism in Liaoning Province, the First Hospital of China Medical University, Shenyang 110001, China

## Abstract

Our previous study showed that Calreticulin (CRT) promoted the development of pancreatic cancer (PC) through ERK/MAPK pathway. We next investigate whether CRT promotes EGF-induced epithelial–mesenchymal transition (EMT) in PC via Integrin/EGFR-ERK/MAPK signaling, which has not been reported yet to our knowledge. EGF simultaneously induced EMT and activated Integrin/EGFR–ERK/MAPK signaling pathway in 3 PC cells. However, CRT silencing significantly inhibited EGF function, including inhibiting EGF-induced EMT-like cell morphology, EGF-enhanced cell invasion and migration, and EGF induced the decrease of E-cadherin, ZO-1, and *β*-catenin and the increase of the key proteins in Integrin/EGFR-ERK/MAPK signaling (pEGFR-tyr1173, Fibronectin, Integrin*β*1, c-Myc and pERK). Conversely, CRT overexpression rescued the change of EMT-related proteins induced by EGF in CRT silencing PC cells. Additionally, CRT was co-stained with pEGFR1173 (with EGF), Fibronectin and Integrin*β*1 by IF under confocal microscopy and was co-immunoprecipitated with Fibronectin, Integrin*β*1 and c-Myc in both PC cells, all of which indicating a close interaction of CRT with Integrin/EGFR–ERK/MAPK signaling pathway in PC. *In vivo*, CRT silencing inhibited subcutaneous tumor growth and liver metastasis of pancreatic tumor. A positive relationship of CRT with Fibronectin, Integrin*β*1, c-Myc and pERK and a negative association of CRT with E-cad was also observed *in vivo* and clinical samples. Meanwhile, overexpression of the above proteins was closely associated with multiple aggressive clinicopathological characteristics and the poor prognosis of PC patients. CRT promotes EGF-induced EMT in PC cells via Integrin/EGFR-ERK/MAPK signaling pathway, which would be a promising therapy target for PC.

From 2000 to 2011, pancreatic cancer (PC) takes up the second upward trend of age-standardized mortality rates in the population of Chinese men.^1^ Strong local invasion and early distant metastasis are the main causes for the worse prognosis of PC, which can be significantly driven by epithelial–mesenchymal transition (EMT).^2^ During EMT, PC loses their epithelial characteristics, gains more invasive and migratory properties of mesenchymal cells and finally contributes to the aggressive progression of PC.^2, 3^

Calreticulin (CRT) is a multi-functional endoplasmic reticulum (ER) protein that regulates a wide array of cellular responses in physiological and pathological processes, including Ca^2+^ homeostasis, transcriptional regulation, immune response and cellular functions (cell proliferation, apoptosis, adhesion and migration, etc).^4, 5^ However, it has pro-tumor or antitumor roles in various cancers depending on its distinct distribution (cell surface, cytoplasm or in the extracellular matrix).^5^ For example, CRT is positively associated with clinical stages, lymph node metastasis and poor prognosis in gastric, breast cancer and esophageal squamous cell carcinoma.^6, 7, 8, 9, 10^ Conversely, reduced CRT expression is observed in malignant effusions of high-grade ovarian carcinoma,^11^ whereas increased CRT expression is associated with better prognosis and differentiated histology in neuroblastoma.^12^ Our previous study showed that CRT overexpression contributed to the development and progression of PC through ERK/MAPK pathway.^13^ ERK/MAPK pathway exhibited a close relationship with Integrin family (a significant regulator in cell migration through enhanced cell–substratum interaction).^14, 15, 16^ Meanwhile, the molecular interactions between Integrin and EGFR-MAPK signaling are prevalent in many cancers,^17, 18, 19^ which has significant roles in the initiation of EMT.^20, 21, 22^ Thus we intend to investigate whether CRT promotes EMT in PC cells via Integrin/EGFR-ERK/MAPK signaling, which has not been reported yet to our knowledge.

## Results

### CRT location and its silencing construction in PC cells

**Figure 1:**
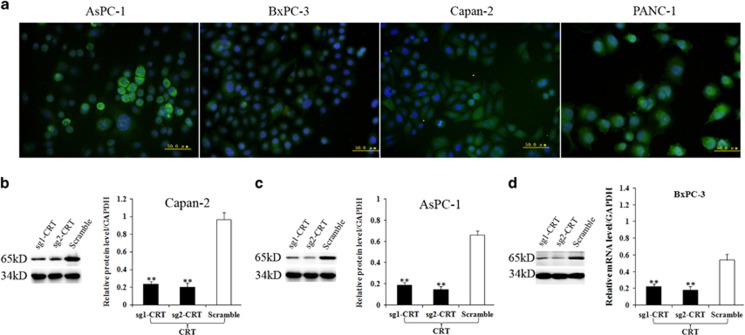
CRT location by IF and its silencing construction in PC cells. (**a**) IF staining of CRT (FITC, green) and nuclear (Hoechst, blue) in 4 PC cell lines. (**b**–**d**) CRT protein level in sg1-CRT, sg2-CRT and scramble-infected Capan-2 (**b**), AsPC-1 (**c**) and BxPC-3 (**d**) cell lines detected by WB. White bars: CRT protein expression in scramble groups. Black bars: CRT protein expression in the sg1-CRT and sg2-CRT groups. **P<0.01 compared with the control

As mentioned above, CRT has a distinct role in cancers partially depending on its intracellular or extracellular location. In line with our previous study,^13^ CRT showed predominantly cytoplasmic expression in four PC cell lines (Figure 1a) by immunofluorescence (IF). Meanwhile, predominant cytoplasmic CRT expression was also observed in clinical tissues by immunohistochemistry (IHC; Figure 10). All of the above indicated predominantly intracellular functions of CRT in PC development. Our previous study showed EGF was much more reliable to induce EMT in AsPC-1, BxPC-3 and Capan-2 cell lines.^3^ Thus above three PC cell lines with relative CRT high expression were used to construct CRT-silencing stable cells via CRISPR/Cas9 system. Western blotting (WB) verified that CRT protein level in Capan-2, AsPC-1 and BxPC-3 cells in the sg1-CRT and sg2-CRT groups were significantly lower than that in the corresponding scramble groups (Figures 1b–d).

### CRT silencing inhibited EGF-induced EMT-like cell morphology, EGF-enhanced cell invasion and migration and EGF-induced the change of EMT-related proteins in two PC cell lines

**Figure 2:**
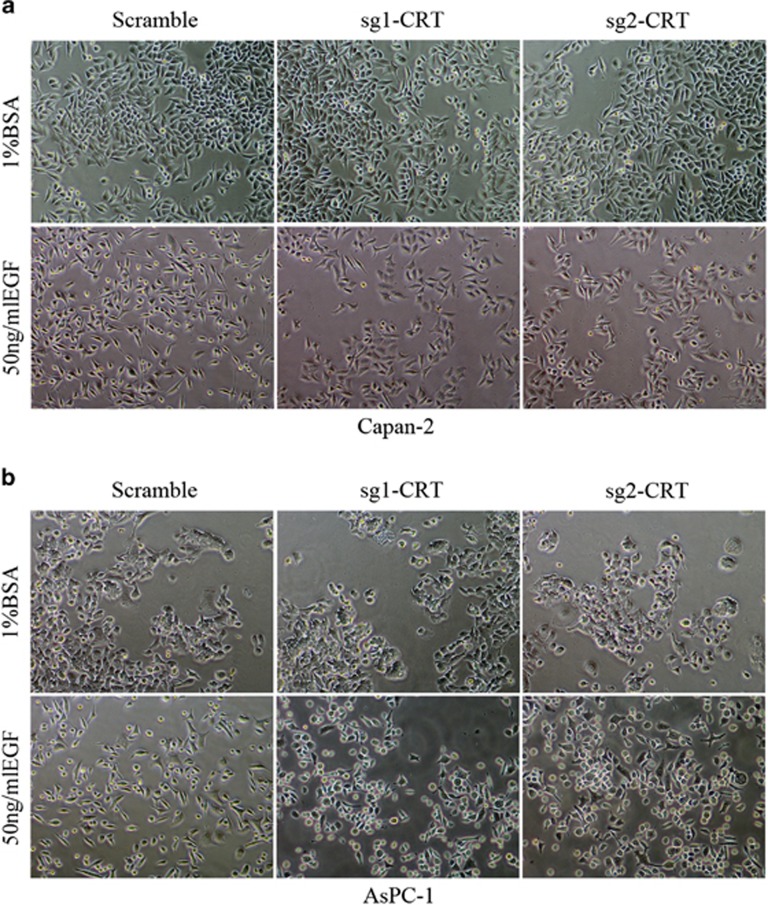
Cell morphology in sg1-CRT, sg2-CRT and scramble-infected Capan-2 and AsPC-1 cells with or without EGF (50 ng/ml) treatment. (**a** and **b**) Under EGF treatment, the fibroblastoid-like phenotype in Capan-2 (**a**) and AsPC-1 (**b**) cells with scramble groups was much more apparent compared with that in the sg1-CRT and sg2-CRT groups

EGF-induced EMT is well studied in our and other previous studies.^3, 23^ Thus we intended to investigate the relationship of CRT with EGF-induced EMT in PC. Upon EGF induction, both Capan-2 and AsPC-1 cells exhibited EMT-like cell morphology: most cells lost their epithelial characteristics and presented a spindle-shaped and fibroblast-like morphology (Figure 2). However, CRT silencing significantly reversed EGF-induced EMT-like cell morphology. Most EGF-treated Capan-2 and AsPC-1 cells restored original cell morphology in the sg1-CRT and sg2-CRT groups. No significant spindle-shaped and fibroblast-like morphology was observed in the sg1-CRT and sg2-CRT groups compared with the corresponding scramble groups (Figure 2). The same results were also observed in BxPC-1 cells (Supplementary Figure 1a).

**Figure 3:**
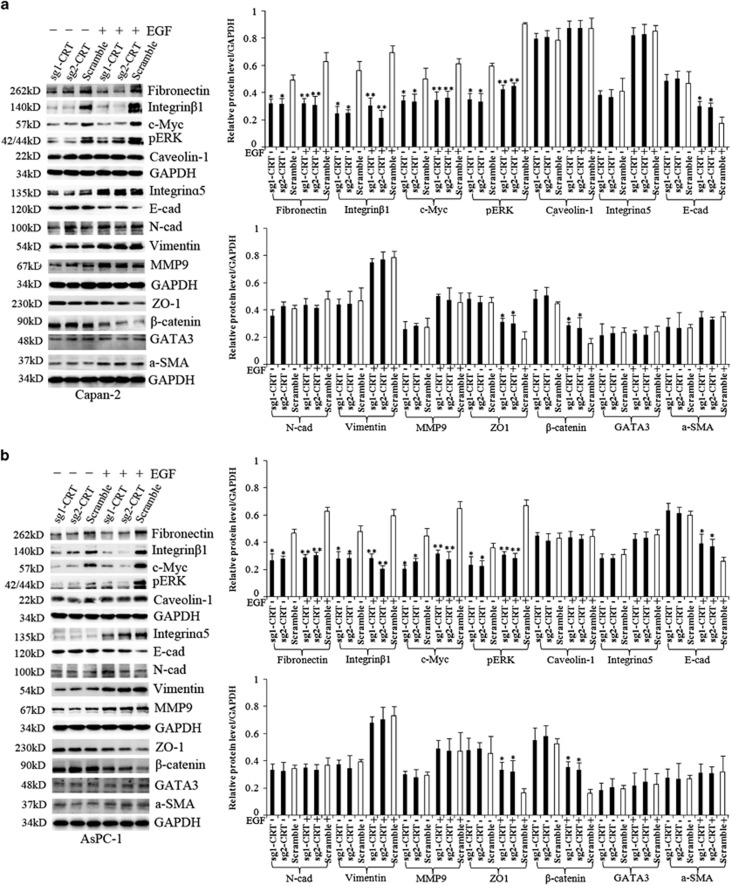
The change of EMT and Integrin/EGFR-ERK/MAPK signaling-related proteins in sg1-CRT, sg2-CRT and scramble-infected (**a**) Capan-2 and (**b**) AsPC-1 cells with or without EGF (50 ng/ml) treatment. White bars: target protein expression in scramble groups with or without EGF treatment. Black bars: target protein expression in the sg1-CRT and sg2-CRT groups with or without EGF treatment. Bars indicate±S.E.*P<0.05; **P<0.01 compared with control

Meanwhile, EGF significantly induced the decrease of E-cad, ZO-1 and *β*-catenin and the increase of Fibronectin, MMP9, Vimentin and a-SMA protein expression (except for N-cad) in both cell lines. However, CRT silencing partially inhibited EGF-induced change of EMT-related proteins, including inhibiting EGF-induced decrease of E-cad, ZO-1 and *β*-catenin and increase of Fibronectin (Figure 3). The same results were also observed in BxPC-1 cells (Supplementary Figure 1b).

**Figure 4:**
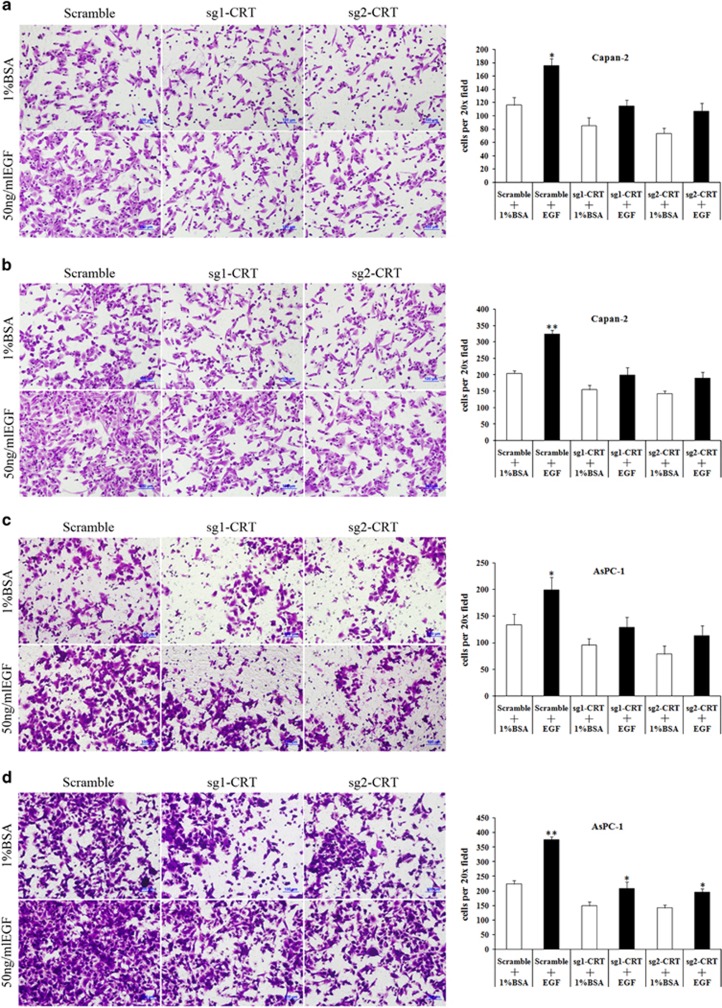
Cell invasion and migration in sg1-CRT, sg2-CRT and scramble-infected PC cells with or without EGF treatment. (**a** and **b**) Cell invasion (**a**) and migration (**b**) in sg1-CRT, sg2-CRT and scramble-infected Capan-2 cells with or without EGF (50 ng/ml) treatment. (**c** and **d**) Cell invasion (**c**) and migration (**d**) in sg1-CRT, sg2-CRT and scramble-infected AsPC-1 cells with or without EGF (50 ng/ml) treatment. Black bars: cell invasion or migration in the scramble, sg1-CRT and sg2-CRT groups with EGF treatment. White bars: cell invasion or migration in the scramble, sg1-CRT and sg2-CRT groups without EGF treatment. Bars indicate±S.E.*P<0.05; **P<0.01 compared with control

It is well known that EMT has a significant role in cell invasion and metastasis of PC.^24^ In current study, EGF significantly enhanced cell invasion and migration in both Capan-2 and AsPC-1 cells (Figure 4). However, CRT silencing inhibited EGF-enhanced cell invasion and migration (Figure 4). Upon EGF induction, a significant increase of cell invasion and migration were found in the scramble groups compared with the corresponding sg1-CRT1 and sg2-CRT groups. The difference of cell invasion and migration in the scramble groups with or without EGF treatment was much more significant than that in the sg1-CRT and sg2-CRT groups (Figure 4). The same results were also observed in BxPC-1 cells (Supplementary Figure 1c).

All together, CRT silencing inhibited EGF-induced EMT in PC cells.

### Overexpression of CRT rescued the change of EMT-related proteins induced by EGF in CRT-silencing Capan-2 cells

**Figure 5:**
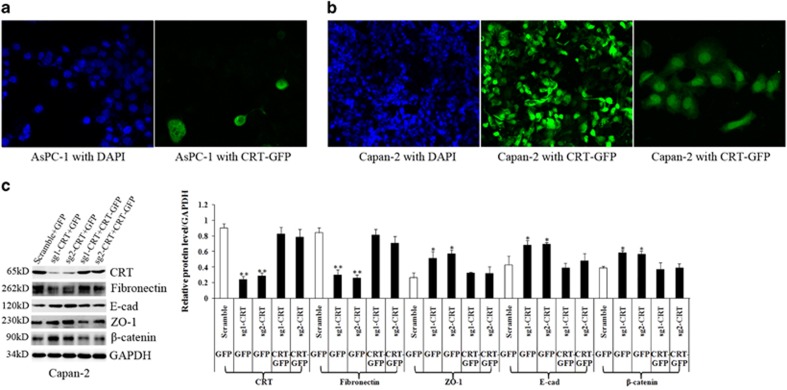
Transfection efficiency of PC cells transfected with CRT-GFP and corresponding rescue experiment. (**a**) AsPC-1 cells showed low transfection efficiency with CRT-GFP. (**b**) Capan-2 cells showed high transfection efficiency with CRT-GFP. (**c**) The change of EMT-related proteins induced by EGF in the sg1-CRT+GFP and sg2-CRT+GFP groups was rescued by CRT ovrerexpression in the sg1-CRT+CRT-GFP and sg2-CRT+CRT-GFP groups. Bars indicate±S.E.*P<0.05; **P<0.01 compared with control

sg1-CRT, sg2-CRT and scramble-infected Capan-2 and AsPC-1 cell lines were transiently transfected with CRT-GFP and GFP plasmids, respectively. Because Capan-2 cells showed much high transfection efficiency compared with AsPC-1 cells, we only used Capan-2 cells for further rescue experiment (Figures 5a and b).

We found that the expression of EMT biomarkers (Fibronectin, E-cad, ZO-1 and *β*-catenin) was rescued by CRT overexpression in sg1-CRT and sg2-CRT-infected Capan-2 cells under EGF treatment (Figure 5c). In detail, the downregulation of E-cad, ZO-1 and *β*-catenin and the upregulation of Fibronectin all induced by EGF in the sg1-CRT+GFP and sg2-CRT+GFP groups was rescued by CRT overexpression in the sg1-CRT+CRT-GFP and sg2-CRT+CRT-GFP groups (Figure 5c). It was well known that membrane-bound CRT is a danger/eat-me signal and important for immunogenic cell death.^25, 26^ However, in the current study, AsPC-1 and Capan-2 cells with CRT-GFP exhibited predominantly cytoplasmic green fluorescence in a perinuclear pattern (but not only on cell surface), which is consistent with the studies by Bibi *et al.*^27^ and by Ihara *et al.*^28^ That might be why high expression of predominantly intracellular CRT executed a pro-tumor role in the development of PC.

### CRT silencing inhibited EGF-activated Integrin/EGFR-ERK/MAPK signaling in two PC cell lines

**Figure 6:**
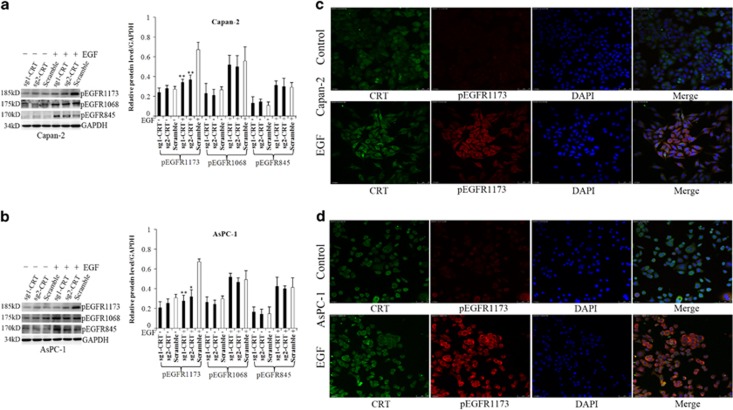
The effect of CRT silencing to pEGFR expression and the co-staining of CRT and pEGFR1173 in PC cells with or without EGF (50 ng/ml) treatment. (**a** and **b**) pEGFR1173, pEGFR1068 and pEGFR845 protein levels in the scramble, sg1-CRT and sg2-CRT groups with or without EGF treatment in Capan-2 (**a**) and AsPC-1 (**b**) cells. (**c** and **d**) Under confocal microscope, the expression and co-staining of CRT and pEGFR1173 in Capan-2 (**c**) and AsPC-1 (**d**) cells by IF with or without EGF (50 ng/ml) treatment. White bars: target protein expression in the scramble group with or without EGF treatment. Black bars: target protein expression in the sg1-CRT and sg2-CRT groups with or without EGF treatment. Bars indicate±S.E.*P<0.05; **P<0.01 compared with control

EGF significantly activated Integrin/EGFR-ERK/MAPK signaling in both Capan-2 and AsPC-1 cells followed by the increase of pEGFR1173, pEGFR1068, pEGFR845, Integrin*β*1, Integrin*α*5, pERK and c-Myc protein expression (Figures 3 and 6). Without EGF, CRT silencing alone did not influence all phosphorylation EGFR expression. However, its silencing significantly inhibited EGF-induced increase of pEGFR1173 but had no effect on EGF-induced pEGFR1068 and pEGFR845 protein levels. Meanwhile, pEGFR1173 was hard to detect and showed no co-staining with CRT by IF without EGF treatment, whereas pEGFR1173 expression was enhanced in cytoplasm and was co-stained with CRT in predominant cytoplasm of Capan-2 and AsPC-1 cells induced by 50 ng/ml of EGF (Figures 6c and d). All the above results implicated a specific interaction of CRT and EGF/EGFR signal pathway.

**Figure 7:**
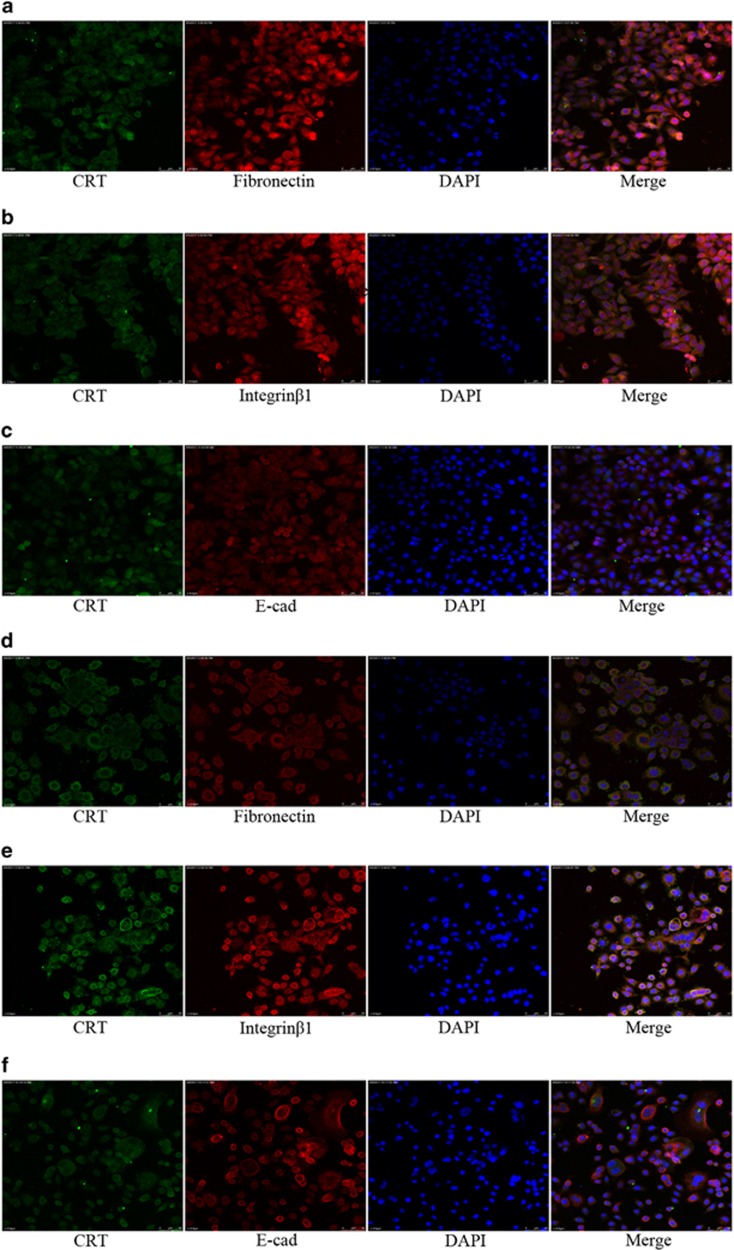
The co-staining of CRT with Fibronectin, Integrin*β*1 and E-cad in PC cells under conforcal microscope. (**a**–**c**) CRT was co-stained with Fibronectin (**a**) and Integrin*β*1 (**b**) in predominant cytoplasm of Capan-2 cells, but not with E-cad (**c**). (**d**–**f**) CRT was co-stained with Fibronectin (**d**) and Integrin*β*1 (**e**) in predominant cytoplasm of AsPC-1 cells, but not with E-cad (**f**)

**Figure 8:**
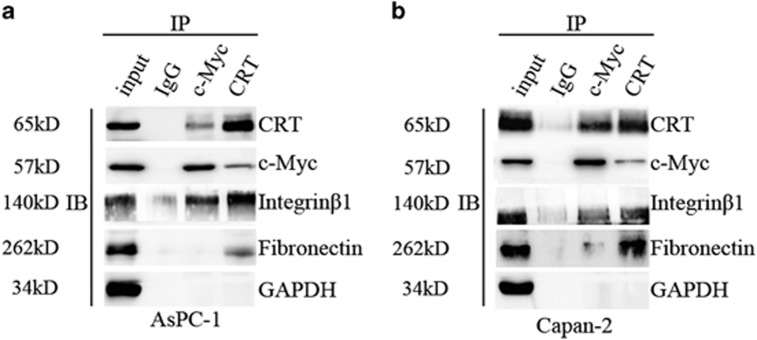
Interaction between CRT, Fibronectin, Integrin*β*1 and c-Myc by immunoprecipitation. (**a**) Capan-2 lysates were immunoprecipitated and WB. (**b**) AsPC-1 lysates were immunoprecipitated and WB. Input and IgG bands were used as positive and negative control, respectively

In addition, CRT silencing alone partially downregulated the key proteins of Integrin/EGFR-ERK/MAPK signaling, including Fibronectin, Integrin*β*1, c-Myc and pERK (except for Integrin*α*5 and caveolin-1) (Figure 3). Meanwhile, both CRT/Fibronectin and CRT/Integrin*β*1 (except for CRT/E-cad) were co-stained in predominant cytoplasm in normal Capan-2 and AsPC-1 cells (Figure 7). Moreover, CRT was co-immunoprecipitated with Fibronectin, Integrin*β*1 and c-Myc in the lysates of normal Capan-2 and AsPC-1 cells (Figure 8). Upon EGF induction, a significant decrease of Fibronectin, Integrin*β*1, pERK and c-Myc were found in the sgCRT1 and sgCRT2 groups compared with the corresponding scramble groups (Figure 3). Taking together, a close interaction between CRT and EGF-induced Integrin/EGFR-ERK/MAPK signaling pathway was observed in PC cells.

### Alteration of intracellular Ca^2+^ mediated by CRT might be involved in regulating EGF-induced EMT and Integrin/EGFR-ERK/MAPK signaling pathway

It is well known that the intracellular Ca^2+^ acting as a second messenger is essential for appropriate cellular functioning, including regulating gene transcription, cell proliferation and migration. Using Fluo-3 to detect intracellular Ca^2+^ under conforcal microscopy, we showed that EGF (50 ng/ml) significantly enhanced intracellular Ca^2+^ just as the function of ionomycin with 100 nmol (a Ca^2+^ ionophore that increased intracellular Ca^2+^ concentrations) in Capan-2 cells (Supplementary Figure 2). Without any stimulus, intracellular Ca^2+^ was partially decreased by CRT silencing, and this trend was much significant under EGF or ionomycin treatment (Supplementary Figure 2). It indicates that the increase of intracellular Ca^2+^ by EGF and ionomycin is finally regulated by CRT. We further showed that Integrin*β*1, Fibronectin and c-Myc proteins were significantly activated by ionomycin in PC cells just as the function of EGF (Supplementary Figure 3). Taking together, we infer that alteration of intracellular Ca^2+^ mediated by CRT has a significant role in EGF-induced EMT and Integrin/EGFR-ERK/MAPK signaling pathway, which will be further investigated in our future study.

### CRT silencing inhibited subcutaneous tumor formation and distant liver metastasis of pancreatic tumor in nude mice

**Figure 9:**
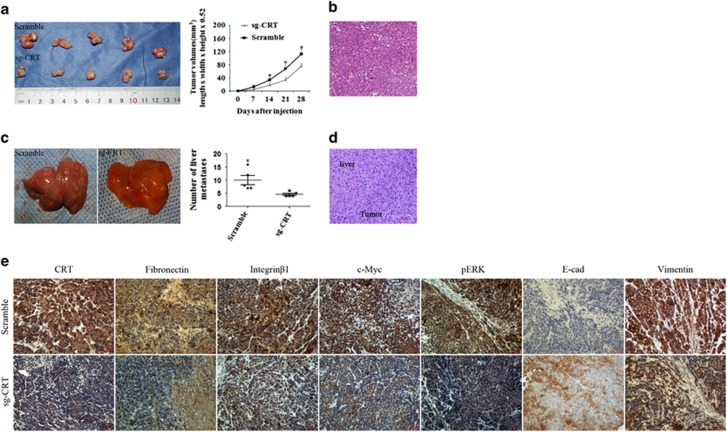
Differences of tumor growth, liver metastasis and the expression of CRT, Fibronectin, Integrin*β*1, c-Myc, pERK, E-cad and Vimentin in the sg-CRT and scramble groups *in vivo*. (**a**) Tumor volumes in nude mice transplanted with sg-CRT and scramble-infected Capan-2 cells. (**b**) The primary tumors were diagnosed by HE staining. (**c**) The average number of liver metastases in nude mice implanted with sg-CRT and scramble-infected AsPC-1 cells. (**d**) Liver metastases were diagnosed by HE staining. (**e**) CRT, Fibronectin, Integrin*β*1, c-Myc, pERK, E-cad and Vimentin expression in subcutaneous pancreatic tumors in the sg-CRT and scramble groups by IHC. Bars indicate±S.E. *P<0.05 compared with control

Capan-2 (derived from primary PC tissue) and AsPC-1 (derived from metastatic ascites) cells were used to construct subcutaneous tumor and distal liver metastasis models, respectively. Tumor volumes in nude mice transplanted with sg-CRT-infected Capan-2 cells were much smaller than that in paired corresponding scramble groups (*P*=0.043) (Figure 9a). The primary tumors were diagnosed under hematoxylin and eosin (HE) staining (Figure 9b). Meanwhile, IHC further verified that CRT, Fibronectin, Integrin*β*1, c-Myc and pERK expression in the sg-CRT groups were much lower than that in the scramble groups (*P*<0.001; *P*<0.01; *P*<0.01; *P*<0.05 and *P*<0.05, respectively), whereas E-cad expression in the sg-CRT groups were much higher than that in the scramble groups using paired sample non-parametric test (Figure 9e). However, Vimentin expression showed no significant difference between the sg-CRT and scramble groups. All above results were consistent with the results *in vitro*.

The average number of liver metastases in nude mice transplanted with sgCRT-infected AsPC-1 cells was much lower than that in the scramble groups (*P*=0.014) (Figure 9c). The distant liver metastases were diagnosed by HE staining (Figure 9d).

### Clinical significance and relationship of CRT with Fibronectin, Integrin*β*1, c-Myc, pERK, E-cad and Vimentin expression levels in PC

Finally, we investigate the close relationship between CRT, Fibronectin, Integrin*β*1, c-Myc and pERK and two classic EMT markers (E-cad and Vimentin) with the clinical significance of PC patients.

**Figure 10:**
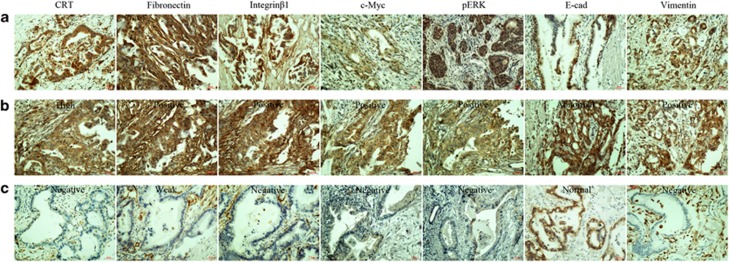
Overexpression of CRT, Fibronectin, Integrin*β*1, c-Myc, pERK, E-cad and Vimentin in PC samples. (**a**) High expression of CRT, positive expression of Fibronectin, Integrin*β*1, c-Myc, pERK and Vimentin and normal E-cad expression in PC tissues. (**b**) The expression of CRT, Fibronectin, Integrin*β*1, c-Myc, pERK, E-cad and Vimentin in one same sample tissue, respectively. (**c**) The expression of CRT, Fibronectin, Integrin*β*1, c-Myc, pERK, E-cad and Vimentin in another one same sample tissue, respectively

The location of CRT in cytoplasm, Fibronectin and Integrin*β*1 in membrane and cytoplasm and c-Myc and pERK in cytoplasm and nuclei were considered for scoring, while the membrane location of E-cad was identified as normal expression (E-cad-negative and cytoplasmic expression were considered as abnormal expression; Figure 10). In 68 cases of PC tissues, proteins were overexpressed in 63.2% (43/68) of CRT, 50% (34/68) of Fibronectin, 69.1% (47/68) of Integrin*β*1, 52.9% (36/68) of c-Myc, 60.2% (41/68) of pERK, 38.2% (26/68) of E-cad and 30.8% (21/68) of Vimentin in IHC assays, respectively (Table 1).

Spearman correlation tests showed that CRT was positively associated with Fibronectin, Integrin*β*1, c-Myc and pERK expression (*P*=0.006; *P*=0.020; *P*=0.008 and *P*=0.036, respectively) and negatively associated with normal E-cad expression (*P*=0.022), but had no association with Vimentin. Using serial sections, PC tissues with high CRT expression was associated with positive Fibronectin, Integrin*β*1, c-Myc, pERK and abnormal E-cad expression levels (Figure 10b) and vice versa (Figure 10c). However, no relationship was found between CRT and Vimentin, although their co-expression was shown in most of PC tissues. All above results were consistent with our observation *in vitro* and *in vivo*.

The clinical significant of c-Myc, pERK, E-cad and Vimentin expression in PC are well studied in other and our previous study.^29, 30, 31, 32^ In the current study, CRT and Fibronectin overexpression had positive association with tumor differentiation, UICC stage and lymph nodes metastasis, respectively, while positive Integrin*β*1 expression was closely associated with UICC stage of PC patients (Table 2).

**Figure 11:**
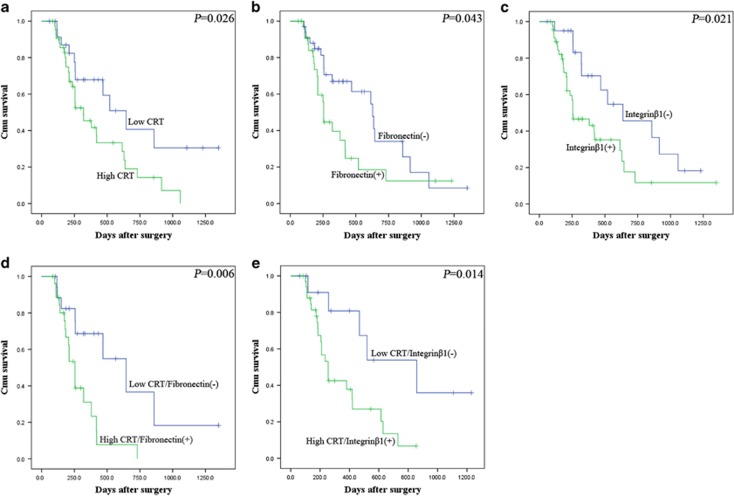
The relationship of CRT, Fibronectin and Integrin*β*1 with the survival of 68 postoperative PC patients in Kaplan–Meier analysis. (**a**) High and low expression of CRT was plotted against overall survival time. (**b**) Positive (+) and negative (−) expression of Fibronectin was plotted against overall survival time. (**c**) Positive (+) and negative (−) expression of Integrin*β*1 was plotted against overall survival time. (**d**) Co-expression of CRT and Fibronectin was plotted against overall survival time. (**e**) Co-expression of CRT and Integrin*β*1 was plotted against overall survival time

In Kaplan–Meier analysis, overexpression of CRT, Fibronectin or Integrin*β*1 contributed to the poor prognosis of PC patients (*P*=0.026; *P*=0.043: *P*=0.021, respectively; Figures 11a–c). Moreover, patients with high CRT and positive Fibronectin expression had a much worse prognosis than in patients with both low/weak expression (*P*=0.006; Figure 11d). This trend was also observed in patients with the co-expression of CRT and Integrin*β*1 (*P*=0.014; Figure 11e). All together, the close interaction of the above proteins coordinately contributed to clinical stage and poor prognosis of PC patients.

## Discussion

Our study first found that CRT silencing inhibited EGF-induced EMT in three PC cell lines. The relationship between CRT and EMT in cancers is rarely reported currently. In gastric cancer cells, TGF-*β*1-induced cell migration and invasion and reciprocal downregulation of E-cad could be abrogated by CRT knockdown.^7^ In MDCK cells, CRT regulates the EMT-like change of cellular phenotype by modulating the Slug/E-cad pathway through altered Ca^2+^ homeostasis.^28^ Our study supplies a new sight to investigate the close interaction of CRT with EGF-induced EMT in PC, which has not been reported to our knowledge. It is well known that EMT (the initial transformation from benign to invasive carcinoma following E-cad decrease) and mesenchymal–epithelial transition (MET, the reverse of EMT in the later metastatic stage with the rescue of E-cad) are recognized as critical events for cancer metastasis.^33, 34^ Overexpression of CRT rescued the change of EMT-related proteins (E-cad, ZO-1, *β*-catenin and Fibronectin) induced by EGF in CRT-silencing PC cells, which further indicates that CRT also has a significant role in EGF-induced MET in PC.

Integrin/EGFR-ERK/MAPK signaling pathway also has a critical role in EMT of various cancers. Inhibition of Fibronectin fibrillogenesis blocks activation of the TGF-*β* signaling pathway via Smad2, decreases cell migration and ultimately leads to inhibition of EMT in colorectal cancer (CRC).^35^ Integrin*β*1 enhances the EMT in association with gefitinib resistance of non-small cell lung cancer.^36^ The activation of integrin*β*1 by type I collagen coupling with the hedgehog pathway promotes EMT in PC.^37^ Both c-Myc expression and MEK1-induced ERK2 nuclear localization are required for TGF-*β*-induced EMT in prostate cancer.^38^ In current study, EGF activated Integrin/EGFR–ERK/MAPK signaling pathway in PC cells. The intreaction between Integrin/EGFR–ERK/MAPK signaling in response to EGF remains controversial. EGF promotes human keratinocyte locomotion on collagen by increasing the integrin*α*5.^39^ EGF has a synergistic effect on EGFR phosphorylation and ERK/MAPK activation in non-small cell lung cancer induced by Integrin*β*1-dependent adhesion.^40^ EGF-induced MAPK signaling inhibits hemidesmosome formation through phosphorylation of the integrin*β*4 in keratinocytes.^41^ Conversely, EGF-mediated Integrin*β*1 inactivation in squamous and colon cancer cell lines was dependent on an EGFR kinase/ERK/p90RSK/filamin A pathway.^42^ Meanwhile, inactivation of integrin a5*β*1 by EGF is correlated with both the level of EGFR expression and p90RSK phosphorylation in squamous carcinoma cells.^43^ Based on our current study, Integrin signaling coordinates with EGF/EGFR–ERK/MAPK pathway in the co-operative control of cell biology as Cabodi *et al.*^44^ and Moro *et al.*^45^ suggest. However, CRT silencing significantly inhibited EGF-induced pEGFR1173, Fibronectin, Integrin*β*1, c-Myc and pERK in PC cells. Moreover, co-staining of CRT with pEGFR1173 (with EGF), Fibronectin and Integrin*β*1 and co-immunoprecipitation of CRT with the key proteins of Integrin/EGFR-ERK/MAPK signaling (Fibronectin, Integrin*β*1 and c-Myc) were detected in PC cells, which is partially observed in Jurkat cells (CRT/integrin *α*2*β*1)^46^ and atria tissues of patients (CRT/integrin*α*5 complex).^47^ In addition, Integrin *α*1*β*1 expression is controlled by c-Myc in CRC cells detected by CHIP assays.^48^ All the above indicated a close interaction of CRT and Integrin/EGFR-ERK/MAPK signal pathway in EGF-induced EMT of PC. We conclude that CRT promotes EGF-induced EMT in PC cells via Integrin/EGFR-ERK/MAPK signaling pathway.

*In vivo*, CRT silencing inhibited subcutaneous tumor growth and liver metastasis of pancreatic tumor, which was consistent with the studies in gastric and bladder cancers.^7, 49^ Moreover, consistent with the results *in vitro*, CRT silencing significantly inhibited Fibronectin, Integrin*β*1, c-Myc and pERK and upregulated E-cad protein expression *in vivo*, which was also observed in clinical tissues. Meanwhile, overexpression of these proteins was closely associated with multiple aggressive clinicopathological characters and poor prognosis of PC patients. Taking together, CRT and Integrin/EGFR–ERK/MAPK signaling pathway coordinately contribute to the aggressive progression of PC.

The corresponding molecular mechanism in the current study is further investigated. With EGF stimulus, CRT co-localized with (bound to) pEGFR1173, directly activated pEGFR and corresponding downstream of ERK/MAPK signaling. Meanwhile, CRT enhanced integrins’ signaling via interacting with Integrin*β*1/Fibronectin. Finally, these two signal pathways coordinately regulated EGF-induced EMT in PC cells as mentioned above.

In addition, CRT might regulate the above signaling via mediating Ca^2+^ homeostasis just as the research in MDCK cells.^28^ Increasing evidences have demonstrated that intracellular alteration of Ca^2+^ homeostasis in various cancer cells is closely involved in tumor initiation, angiogenesis, progression and metastasis.^50^ In the current study, the increase of both intracellular Ca^2+^ and Integrin*β*1, Fibronectin and c-Myc expression induced by EGF and ionomycin is finally regulated by CRT. CRT also couples Ca^2+^ release and Ca^2+^ influx in integrin-mediated Ca^2+^ signaling.^51, 52^ A positive modulation of ERK/MAPK signaling pathways by an increase in intracellular Ca^2+^ is also well reported in neurons and PC12 cells after stimulation with neurotrophins or growth factors.^53, 54^ The Ca^2+^ sensor calmodulin enhance Myc transcriptional and oncogenic activities in Ca^2+^-dependent manner.^55^ Additionally, the interaction of Integrin/Fibronectin and ERK/MAPK pathway is always mediated by Ca^2+^ signaling. In immortalized thyroid TAD-2 cells, activation of the integrin receptors by Fibronectin binding stimulates the Ras/ERK pathway, which is controlled by calcium calmodulin-dependent kinase II-mediated Ca^2+^ signaling.^56^ In normal thyroid cells, both induction of pERK and increase of intracellular Ca^2+^ were mediated by Fibronectin binding to integrin *α*v*β*3.^57^

Taking together, CRT promotes EGF-induced EMT in PC cells via Integrin/EGFR-ERK/MAPK signaling pathway, which might be regulated by CRT-mediated alteration of intracellular Ca^2+^. Recently, it remains unclear how ER or non-ER CRT functions are stimulated in different cancers. All of the above will be further investigated in our future study.

## Materials and methods

### Tissue samples

All patient-derived specimens were collected and archived under protocols approved by the institutional review board of China Medical University. Sixty-eight cases of paraffin-embedded pancreatic ductal adenocarcinoma were obtained from the patients at the First Hospital of China Medical University between 2011 and 2016. All diagnoses were confirmed pathologically. Patients with endocrine carcinoma, acinar cell carcinoma and invasive intraductal papillary mucinous carcinoma were excluded from this study. Patients’ characteristics were summarized in Supplementary Table 1.

### Cell lines and culture

AsPC-1, BxPC-3 and PANC-1 human PC cell lines were purchased from the Cell Bank of the Chinese Academy of Sciences (Shanghai, China). Capan-2 cells were obtained from the American Type Culture Collection (ATCC, Manassas, VA, USA). These cell lines were maintained in the recommended growth media with 10% fetal bovine serum (FBS; Hyclone, Logan, UT, USA).

### IF staining

Briefly, PC cells were plated in 24-well culture plates overnight, fixed in 4% paraformaldehyde for 30 min, permeabilized with 0.2% Triton X-100 for 10 min, incubated with 5% BSA for 2 h and then stained for the primary antibodies: monoclonal anti-CRT antibody (Abcam, Cambridge, UK), CRT/pEGFR-Tyr1173 (pEGFR1173, Abcam), CRT/Fibronectin (Proteintech, Chicago, IL, USA), CRT/Integrin*β*1 (Abcam) and CRT/ E-cadherin (E-cad, Abcam) in 4 °C overnight. The secondary antibodies were conjugated with green FITC (Proteintech) and red TRIC (Proteintech). Cells were then co-stained with Hoechst or DAPI (Sigma, St. Louis, MO, USA) for nuclei visualization and finally viewed by fluorescence microscope (Nikon Microphot-FX, Tokyo, Japan) and confocal fluorescence microscopy (Leica Tcs Sp5 II, Leica, Heidelberg, Germany).

### Immunohistochemistry

IHC was performed as described previously.^13, 58^ Staining intensity was scored as 0 (negative), 1 (weak), 2 (medium) and 3 (strong). Extent of staining was scored as 0 (0%), 1 (1–25%), 2 (26–50%), 3 (51–75%) and 4 (76–100%) according to the percentage of the carcinoma involved area that was positively stained. The final IHC staining scores were determined by three professional pathologists. The sum of the extent and intensity score was used to arrive at the final staining scores (0–7). For CRT, tumors with a final staining score of >3.5 were considered as high expression. For Fibronectin, Integrin*β*1, c-Myc, pERK, E-cad and Vimentin, tumors with a final staining score of ≥2 were considered as positive expression. For E-cad, membrane expression in tumors with a final staining score of ≥2 was identified as normal expression; whereas negative and cytoplasmic E-cad expression was considered as abnormal expression. We used the same score methods to analyze the IHC assays both in *in vivo* and clinical samples.

### Western blotting

For WB, Samples were loaded onto 10% SDS-polyacrylamide gels, transferred to polyvinylidene difluoride membranes (Millipore Corp, Bedford, MA, USA) and incubated with primary CRT, pEGFR1173, pEGFR-Tyr1068 (pEGFR1068) (Abcam), pEGFR-Tyr845 (pEGFR845) (Abcam), Fibronectin, Integrin*β*1, Integrin*α*5 (Abcam), c-Myc (Cell Signaling Technology, Danvers, MA, USA), pERK (Cell Signaling Technology), Caveolin-1 (Proteintech), E-cad, N-cadherin (N-cad, Abcam), Vimentin (Proteintech), MMP9 (Proteintech), ZO-1 (Proteintech), *β*-catenin (Proteintech), GATA3 (Proteintech), alpha smooth muscle actin (a-SMA, Abcam) and GAPDH (Proteintech) antibodies overnight at 4 °C. Membranes were incubated with horseradish peroxidase-conjugated monoclonal secondary antibody (Santa Cruz, CA, UK) at room temperature for 1.5 h, respectively. Immunoreactive protein bands were visualized with an ECL Detection Kit (Thermo scientific, Rockford, IL, USA). Each experiment was repeated three times.

### Immunoprecipitation

For immunoprecipitation, as described previously ^58^, whole protein lysates (prepared from Capan-2 and AsPC-1 cells) were extracted in a lysis buffer (20 mM Tris/HCl, pH7.4, 1.0% NP-40, 1 mM EDTA, 150 mM NaCl, 50 μg/ml PMSF, 10 μg/ml leupeptin). Briefly, CRT, c-Myc and IgG (Santa Cruz) antibodies were preincubated with magnetic beads (Bio-Rad, Hercules, CA, USA) for 4 h at 4 °C. The antibody–beads complex was washed three times with the lysis buffer and incubated with the soluble supernatants of protein lysates overnight at 4 °C. Next, the immunocomplexes were washed three times with lysis buffer, eluted by boiling in sample loading buffer for SDS-PAGE and then subjected to WB analysis with CRT, c-Myc, Integrin*β*1, Fibronectin and GAPDH antibodies. Each experiment was repeated three times.

### Construction of CRT-silencing stable cell lines using CRISPR/Cas9 and transient transfection for rescue experiment

Lenti-cas9 and Lenti-sgRNA were synthesized from Genechem (GenePharma Co, Ltd, Shanghai, China). Cells were firstly infected with lenti-cas9 in AsPC-1 and Capan-2 cell lines and next selected by puromycin (Sigma). The stable sublines were then infected with CRT-sgRNA (sg1-CRT/sg2-CRT) and sgRNA control (scramble) to specifically silence target genes. The target sequences of sg1-CRT, sg2-CRT and scramble are shown in Supplementary Table 2. CRT-silencing effect was successfully verified by WB shown in ‘Results section’ (Figure 1). The PCMV2-CRT plasmid (CRT-GFP) and corresponding empty plasmid (GFP) were gifts from Dr. Hassan Dihazi as described previously.^27^ sg1-CRT, sg2-CRT and scramble-infected PC cells with EGF treatment were transiently transfected with CRT-GFP and GFP plasmids for 72 h using oligofectamine3000 (Invitrogen, Carlsbad, CA, USA) as described by the manufacturer. Transfection efficiency was detected by confocal fluorescence microscopy.

### EMT construction

According to our previous study,^3^ sg1-CRT, sg2-CRT and scramble infected three PC cell lines (Capan-2, AsPC-1 and BxPC-3 cells) were treated with 50 ng/ml EGF (Peprotech, RockyHill, NJ, USA) twice within 48–72 h. 1% BSA (Sigma) was used as a control. Cells were cultured with recommended growth media containing 1% FBS in order to enhance the effect of EGF. The formation of EMT was verified by the observation of EMT-like cell morphology (a spindle-shaped and fibroblast-like morphology), EMT-induced cell invasion and migration and the change of EMT-related proteins.

### Invasion and migration assays

Cell invasion was assessed with modified boyden chamber (BD Biosciences, Sparks, MD, USA) assays. Briefly, sg1-CRT, sg2-CRT and scramble-infected PC cells with or without EGF (50 ng/ml) treatment was seeded onto 8.0-μM pore size membrane inserts coated with matrigel (BD Biosciences) in 24-well plates with FBS-free growth media. Growth media plus 10% FBS was added to the bottom wells with or without EGF treatment as a chemoattractant. After 24 h, cells that did not migrate were removed from the top side of the inserts with a cotton swab. Cells that had migrated to the underside of the inserts were stained with Crystal Violet Hydrate (Sigma) according to the manufacturer’s instructions. The migration assay was carried out in a similar manner without matrigel. The migratory cells were counted under a microscope at × 20 magnification. Cell images were obtained using a microscope (Nikon Microphot-FX). Cells were counted in five random fields per insert. Results are expressed as cells migrated per field.

### *In vivo* xenograft model

Animals were maintained according to institutional regulations in facilities approved by the Animal Care Committee of China Medical University in accordance with Chinese government guidelines for animal experiments.

Bilateral axillas injection was used to construct subcutaneous tumor formation, while tail vein injection was used to construct distant liver metastasis model.

A total of 15 cases of nude mice (BALB/c-nu) were acclimatized for a week. sg-CRT and scramble-infected Capan-2 cells (5 × 10^6^/ml) suspended in 200 *μ*l of FBS-free 1640 were subcutaneously transplanted into bilateral axillas of five mice, respectively. A cotton swab was used to avoid possible bleeding and leakage of tumor cells from the injection site. The mice were killed 4 weeks later. Tumors were excised and documented by measurements using vernier calipers. Tumor volumes were calculated by the following formula: length × width × height × 0.52 in millimeters. Finally, samples were extracted and fixed for late HE and IHC staining.

sg-CRT and scramble-infected AsPC-1 cells (1 × 10^7^/ml) suspended in 100 *μ*l of FBS-free 1640 were injected into the tail vein of 10 cases of nude mice, respectively. A cotton swab was held over the injection site for 1 min to prevent leakage from tail vein. The mice were killed 8 weeks later. The number of liver metastases was investigated immediately and then fixed for HE and staining.

### Statistical analysis

Statistical analyses were performed using the SPSS software 13.0 (SPSS, Chicago, IL, USA). The differences of orthotopic tumor volumes were compared by paired sample *t*-test. The differential expression of target proteins by IHC in orthotopic tumor were compared through paired sample non-parametric test. The differences of WB analysis, cell migration and invasion assays and of the number of liver metastases were expressed as mean±S.E. and compared by Student’s *t*-test. The clinicopathological significance of and relationship between target proteins were analyzed by Chi-squared and Spearman correlation tests, respectively. Kaplan–Meier curve was used to estimate survival, and differences were analyzed by log-rank test. A value of *P*<0.05 indicated statistical significant.

## Publisher’s Note

Springer Nature remains neutral with regard to jurisdictional claims in published maps and institutional affiliations.

## Figures and Tables

**Table 1:** Relationship of CRT with Fibronectin, Integrin*β*1, c-Myc, pERK, E-cad and Vimentin

**Parameters**		**CRT**		**Spearman rank**	***P***
		**Low (25)**	**High (43)**		
Fibronectin	Negative (34)	18	16	0.335	0.006
	Positive (34)	7	27		
Integrin*β*1	Negative (21)	12	9	0.283	0.020
	Positive (47)	13	34		
c-Myc	Negative (32)	17	15	0.320	0.008
	Positive (36)	8	28		
pERK	Negative (27)	14	13	0.254	0.036
	Positive (41)	11	30		
E-cad	Abnormal (42)	11	31	−0.279	0.022
	Normal (26)	14	12		
Vimentin	Negative (47)	19	28	0.114	0.349
	Positive (21)	6	15		

**Table 2:** Association of CRT, Fibronectin and Integrin*β*1 expression levels with clinical data

**Parameters**	**No. of patients**	**CRT**		***P***	**Fibronectin**		***P***	**Integrin*****β*****1**		***P***
		**Low**	**High**		**Negative**	**Positive**		**Negative**	**Positive**	
Cases	68									

*Age(years)*										
≤65	54	20	34	0.927	29	25	0.230	18	36	0.593
>65	14	5	9		5	9		3	11	
										
*Gender*										
Male	47	18	29	0.695	23	24	0.793	12	35	0.153
Female	21	7	14		11	10		9	12	

*Tumor location*										
Head	49	17	32	0.570	25	24	0.787	18	31	0.093
Body–tail	19	8	11		9	10		3	16	
										
*Tumor size (cm)*										
<2.5	21	10	11	0.215	12	9	0.431	9	12	0.153
≥2.5	47	15	32		22	25		12	35	
										
*Differentiation*										
Well	24	12	12	0.095	16	8	0.042	10	14	0.155
Moderate and poor	44	13	31		18	26		11	33	

*T stage*										
T1+T2	22	10	12	0.304	14	8	0.120	8	14	0.499
T3+T4	46	15	31		20	26		13	33	
										
*Lymph nodes metastasis*										
N0 (negative)	53	23	30	0.033	30	23	0.041	19	34	0.096
N1 (positive)	15	2	13		4	11		2	13	
										
*UICC stage*										
I+IIA	50	22	28	0.039	29	21	0.028	19	31	0.034
IIB+III	18	3	15		5	13		2	16	
										
*Perineural invasion*										
Absent	47	19	28	0.349	26	21	0.189	17	30	0.158
Present	21	6	15		8	13		4	17	
										
*Vascular permeation*										
Absent	37	15	22	0.481	22	15	0.088	14	23	0.175
Present	31	10	21		12	19		7	24	
										
*Pretherapeutic CA19-9 level*										
<37 U/ml	16	8	8	0.209	9	7	0.567	7	9	0.203
≥37 U/ml	52	17	35		25	27		14	38	
