# Supplementary figures and images for: Calreticulin promotes EGF-induced EMT in pancreatic cancer cells via Integrin/EGFR-ERK/MAPK signaling pathway
Source: Cell Death Dis. 2017 Oct 26;8(10):e3147–. doi: 10.1038/cddis.2017.547 (PMC5680916; doi:10.1038/cddis.2017.547)

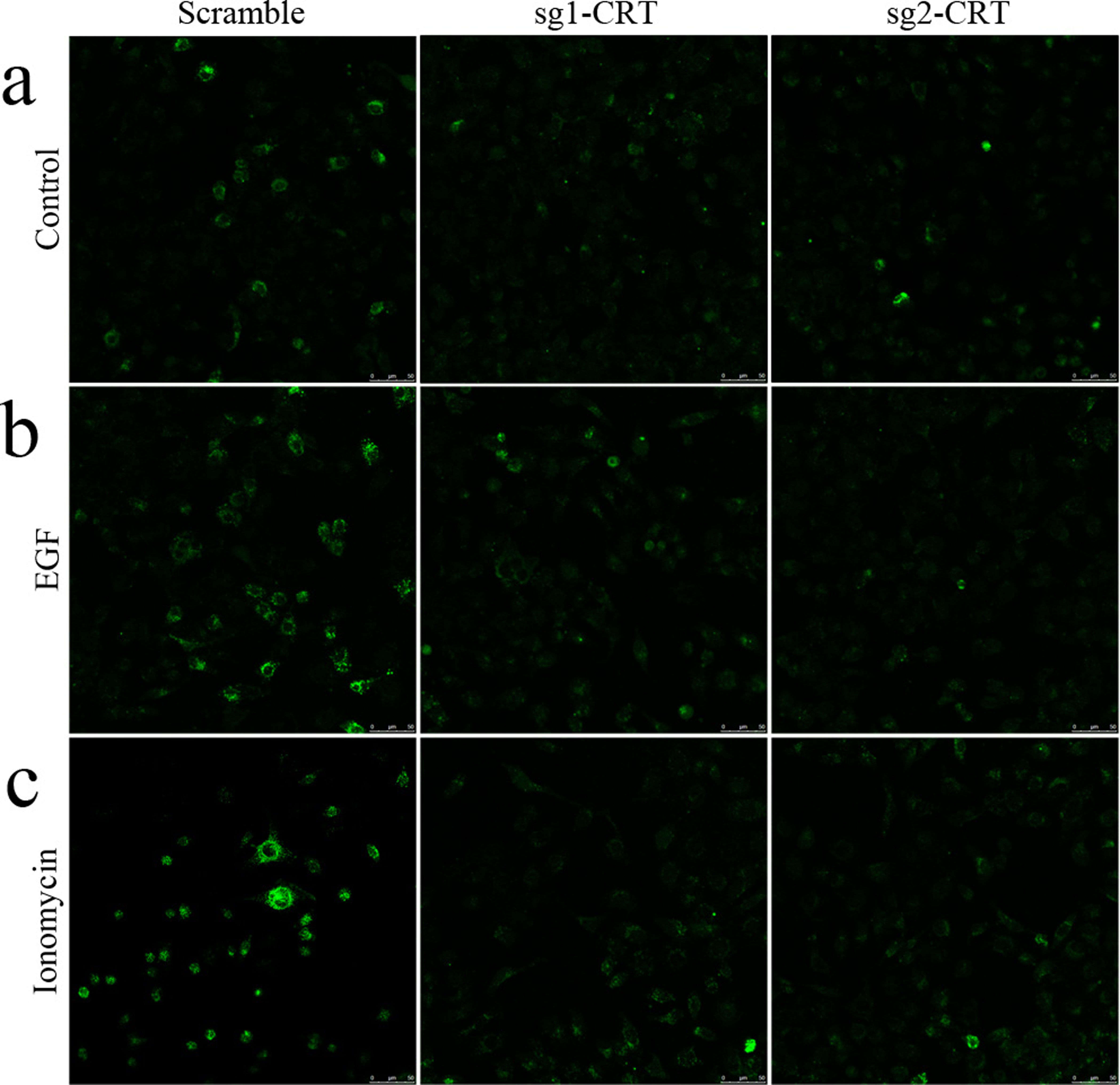

Supplement: Supplementary Figure 2 [file cddis2017547x2.tif]

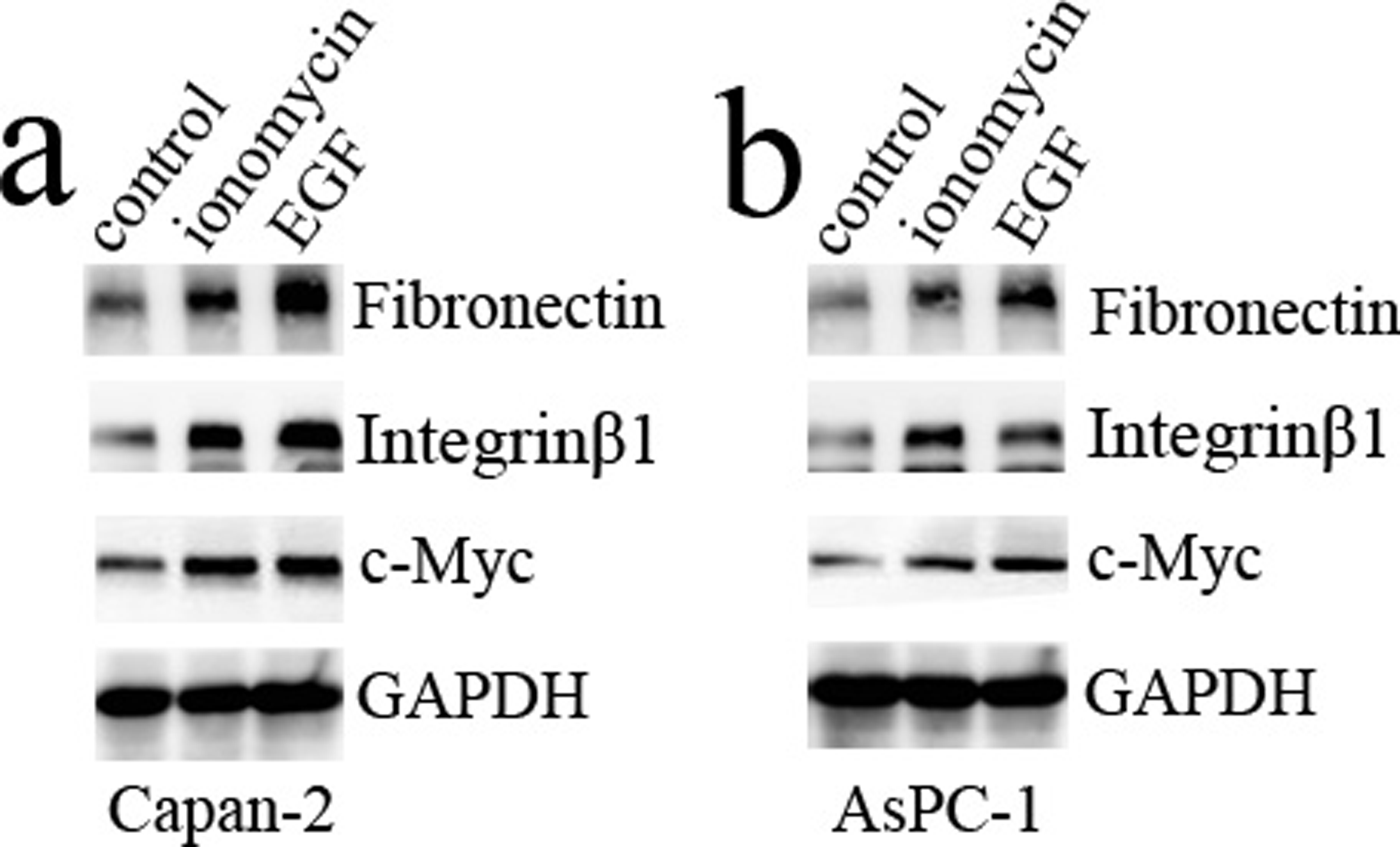

Supplement: Supplementary Figure 3 [file cddis2017547x3.tif]
