# Supplementary material for: Calreticulin promotes EGF-induced EMT in pancreatic cancer cells via Integrin/EGFR-ERK/MAPK signaling pathway
Source: Cell Death Dis. 2017 Oct 26;8(10):e3147–. doi: 10.1038/cddis.2017.547 (PMC5680916; doi:10.1038/cddis.2017.547)
Supplement: Supplementary Table 1 [file cddis2017547x4.docx]

**Supplemental Material Table 1**. Clinical data of 68 PC patients

| Parameters | No. of patients |
| --- | --- |
| Cases | 68 |
| Age(years) |  |
| ≤65 | 54 |
| >65 | 14 |
| Gender |  |
| Male | 47 |
| Female | 21 |
| Tumor location |  |
| Head | 49 |
| Body-tail | 19 |
| Tumor size(cm) |  |
| <2.5 | 22 |
| ≥2.5 | 46 |
| Differentiation |  |
| Well | 24 |
| Moderate and poor | 44 |
| T stage |  |
| T1+T2 | 22 |
| T3+T4 | 46 |
| Lymph nodes  metastasis |  |
| N0(negative) | 53 |
| N1(positive) | 15 |
| UICC stage |  |
| I+IIA | 50 |
| IIB+III | 18 |
| Perineural invasion |  |
| Absent | 47 |
| Present | 21 |
| Vascular permeation |  |
| Absent | 37 |
| Present | 31 |
| Pre-therapeutic CA19-9 level |  |
| <37 U/ml | 16 |
| ≥37 U/ml | 52 |
