# Supplementary material for: Calreticulin promotes EGF-induced EMT in pancreatic cancer cells via Integrin/EGFR-ERK/MAPK signaling pathway
Source: Cell Death Dis. 2017 Oct 26;8(10):e3147–. doi: 10.1038/cddis.2017.547 (PMC5680916; doi:10.1038/cddis.2017.547)
Supplement: Supplementary Table 2 [file cddis2017547x5.docx]

Supplemental Material Table 2. The target sequences of sg-CRT1, sg-CRT2 and scramble.

| Gene | Oligo Name | Oligo Sequence |
| --- | --- | --- |
| CRT | sg-CRT1 | －: GAAGATGACATGAACCTTCT |
|  | sg-CRT2 | +: CGAGCCTGCCGTCTACTTCA |
| NC | Scramble | TTCTCCGAACGTGTCACGT |
