# Supplementary material for: Calreticulin promotes EGF-induced EMT in pancreatic cancer cells via Integrin/EGFR-ERK/MAPK signaling pathway
Source: Cell Death Dis. 2017 Oct 26;8(10):e3147–. doi: 10.1038/cddis.2017.547 (PMC5680916; doi:10.1038/cddis.2017.547)
Supplement: Supplementary Figure Legends [file cddis2017547x6.docx]

Supplemental Material Fig 1. The role of CRT in EGF induced EMT in BxPC-3 cells. **a** Cell morphology in sg1-CRT, sg2-CRT and scramble infected BxPC-3 cells with or without EGF (50ng/ml) treatment. **b** The change of EMT and Integrin/EGFR-ERK/MAPK signaling related proteins in sg1-CRT, sg2-CRT and scramble infected BxPC-3 cells with or without EGF (50ng/ml) treatment. **c and d** Cell invasion (c) and migration (d) in sg1-CRT, sg2-CRT and scramble infected BxPC-3 cells with or without EGF treatment. Bars indicate ± S.E.*, *P* <0.05; **, *P* <0.01 compared with the control.

Supplemental Material Fig 2. Intracellular Ca^2+^ level in scramble, sg1-CRT, and sg2-CRT groups with or without EGF and ionomycin treatment using Fluo-3 under confocal microscopy. Both EGF (50ng/ml) and ionomycin (100nmol) significantly enhanced intracellular Ca^2+^. Without stimulus, intracellular Ca^2+^ was partially decreased by CRT silencing, and this trend was much significant when under EGF or ionomycin treatment.

Supplemental Material Fig 3. Increase of intracellular Ca^2+^ induced by ionomycin (100nmol) and EGF (50ng/ml) activated Integrinβ1, Fibronectin and c-Myc protein expression in Capan-2 (a) and AsPC-1 (b) cells.
